# Silence, Awkwardness and Discomfort: Understanding How Health Interventions Fail—An Ethnographic Study of a Peer Support Intervention for People With Type 2 Diabetes in Denmark

**DOI:** 10.1111/1467-9566.70088

**Published:** 2025-09-27

**Authors:** Astrid Andrea Anesen, Bryan Cleal, Tine M. Gammeltoft

**Affiliations:** ^1^ Department of Anthropology University of Copenhagen Copenhagen Denmark; ^2^ Department of Prevention Health Promotion, and Community Care Copenhagen University Hospital—Steno Diabetes Center Copenhagen Herlev, Capital Region Denmark

## Abstract

Unsuccessful peer support interventions rarely receive detailed ethnographic attention. The article examines a peer support intervention part of a randomised controlled trial designed to provide people with type 2 diabetes socio‐emotional support while adopting self‐care technology. The trial concluded that the intervention yielded no benefits. This article insists on ‘failure’ as an analytical opening with potential for intervention research. Ethnographic fieldwork was conducted in Denmark between January 2020 and June 2022. The ethnographic material comprises intervention audio recordings and observations involving 15 participants, as well as follow‐up interviews. Using the concept of ‘lifeworld’ to understand participants' perspectives and ‘the will to improve’ to interpret the intervention's framework, we unpack the tensions and awkwardness that permeated meetings. Comprehending why the intervention's activities failed to engage participants meaningfully, we highlight how it framed diabetes as a socio‐emotional problem in need of fixing, thereby problematising living with the illness, contrasting participants' shared endeavours to de‐problematise the experience within the intervention. The intervention offered a version of their lives with values and priorities that did not resonate and was thereby unsuccessful in bringing the aimed‐for outcomes. Challenging the will to improve, we propose the ‘lifeworlding’ of future health interventions, prioritising lived experiences in the very framing of interventions.

## 1 Introduction

### 1.1 The Shaping of an Intervention: Addressing Socio‐Emotional Needs

Diabetes can significantly affect people's lives. Peer support interventions are increasingly utilised to support people with chronic illnesses, such as type 2 diabetes (T2D), manage daily self‐care (Brownson and Heisler 2009; Garn et al. 2021). Living with diabetes implies recognising that the body no longer works predictably, and one can no longer rely on it to adjust glucose levels automatically. Diabetes thus requires constant decision‐making regarding diet, exercise and medication to stabilise glucose levels, making self‐care crucial (Davis et al. 2022). In biomedical terms, continuous glucose monitoring (CGM) may improve glycaemic, metabolic and behavioural outcomes for people with T2D (Taylor et al. 2018). CGM consists of a skin‐attached sensor transmitting real‐time glucose values via Bluetooth to an app or a manufacturer‐provided device. CGM triggers audible alarms when glucose levels are high or low (Dexcom 2025). CGM helps people make informed self‐care decisions and can replace traditional glucose monitoring, which requires pricking a finger to measure blood glucose. Yet, managing diabetes with new technologies can be complex (Davies et al. 2022), leaving many people feeling isolated and in need of social support (Hansen et al. 2015; Hempler et al. 2016; Davis et al. 2022). Peer support can offer social, emotional and practical assistance in adopting and maintaining self‐care (Dale et al. 2012; Fisher et al. 2017).

This qualitative article reports on a Danish randomised controlled trial (RCT) examining a peer support intervention for people with T2D new to CGM, hypothesised to address socio‐emotional challenges and optimise its use. Participants were randomised into three groups: (A) CGM alone, (B) CGM with peer support and (C) standard care. Compared to baseline (measured for the total CGM group), both CGM groups experienced improvements in glycaemic (e.g., HbA1c), metabolic (e.g., BMI), and patient‐reported outcome measures (e.g., questionnaire on well‐being) (see Lind et al. 2024 for further information). Despite this relative success, the RCT found no added benefits from the peer support component when evaluated by those measures, indicating a positive effect of CGM but not of peer support. The RCT concluded that there is a need to understand this lack of impact from participants' perspectives, to unpack how the peer support intervention failed to improve mental and physical health (Lind et al. 2024).

### 1.2 Rethinking Peer Support Research: An Ethnographic Approach

Studies have suggested that the evidence for peer support interventions is limited and inconsistent (Chen et al. 2024; Dale et al. 2012). Furthermore, existing research often highlights the potentials (Egmose et al. 2024; Fisher et al. 2017; Mikolajczak‐Degrauwe et al. 2023), while the challenges have received little attention (Chien et al. 2006; Coreil et al. 2004; Joo et al. 2022). Some studies have examined barriers to implementing group‐based peer support for people with diabetes using questionnaires (Aziz et al. 2018). Such methods have difficulties capturing lived experiences. Garn et al. (2021) underscored the importance of participants' social contexts in understanding barriers to one‐on‐one peer support interventions for vulnerable people with diabetes; however, this study relied solely on questionnaires. It has been suggested that peer support research needs to be reconsidered, adopting different measures and methodologies that take participants' perspectives seriously (Walker and Peterson 2021). Therefore, to understand why health interventions ‘succeed’ or ‘fail’, we argue, requires alternative research approaches prioritising lived experiences.

A study using visual‐based narrative research methods to explore participants' experiences with peer support for people with diabetes found that these primarily captured positive experiences (Pienaar and Reid 2021), making it challenging to uncover underlying social mechanisms. Because peer support operates within a complex system involving people's responses to illness, society, the healthcare system and broader social structures (Walker and Peterson 2021), ethnographic methods may help rethink health interventions as social phenomena. However, peer support is rarely studied ethnographically (see Coreil et al. 2004; Frigerio and Montali 2016; Lasanen et al. 2017 for exceptions). This article examines how people with diabetes experienced a peer support intervention that failed to meet expectations, as measured by both biomedical and patient‐reported outcome measures. Whereas much attention has been paid to how health interventions are designed and what they offer, we focus on how such interventions are lived, locally negotiated and sometimes resisted by the people for whom they are intended. Inspired by Kirkegaard (2022), we provide empirically driven insights into the social dynamics of the intervention through an ethnographic lens. Following Gibson et al. (2022), we consider participants' experiences and draw on the rich ethnographic literature on living with diabetes (Gammeltoft 2024; Mendenhall 2019; Moran‐Thomas 2019). From that point of departure, we explore participants' perspectives on the challenges that emerged during its implementation.

### 1.3 Analytical Focus: Lived Experiences of Health Interventions

We frame our analysis through the dual lens of ‘lifeworld’ and ‘the will to improve’. Together, the concepts help illuminate what occurred in the meeting between a professionally led health intervention and its target population, or, phrasing it phenomenologically, they help serve to illustrate that ‘there is always an irreducible asymmetry and instability of perspectives and experiences assumed in even the most mutually attuned, empathic and intimate of intersubjective encounters’ (Desjarlais and Throop 2011, 91).

In phenomenology, the concept of lifeworld is central to the study of experience. Husserl (1970) first defined the lifeworld (*Lebenswelt*) as the mundane world of our everyday lives as it appears before us. It is the world we live in, before any theoretical analysis or abstractions, in which human meaning occurs through our perceptions and experiences. Drawing on Husserl's concern that science had become detached from the textures of lived experience, and Schutz’s (1967) subsequent introduction of the lifeworld into sociological thought and other empirical traditions, we foreground the everyday taken‐for‐granted world in which the intervention was embedded. Rather than treating context as a backdrop, we employ the lifeworld perspective to highlight how peer support was interpreted and enacted within the rhythms and constraints of participants' daily lives. This lens is central to our argument: that successful implementation cannot be understood apart from the subjective and social worlds of those involved.

We anchor our conceptual framework in embodiment theory and phenomenology, tying illness to its experience and people's bodies to their lifeworlds. The concept of lifeworld has been frequently employed in medical anthropology to understand how people cope with illness (Biehl et al. 2007; Good 1993; Jackson 2012; Kleinman 1988). Good (1993) notes about the experience of illness, ‘the body as physical object and as agent of experience did not belong to separate worlds. The illness was present in the lived body. It was experienced as a change in the lifeworld’ (117). Drawing on Good's thoughts, we address the body not only as an object for intervention but also as a site of lived experience. In other words, we treat the body as a source of experience. Lifeworld has also been utilised to understand health interventions (Hemingway 2011; Russo and Kelly 2023). We build on these studies to explore a peer support intervention embedded in the lifeworlds of people with diabetes.

Complementing this, we employ Li’s (2007) notion of the will to improve, developed in Indonesia, which captures the underlying rationalities of health interventions. According to Li, expert interventions that guide people towards better ways of living exercise power in the form of Foucauldian ‘governmentality’. Expert interventions aim to improve people's lives by ‘educating desires and configuring habits, aspirations and beliefs’ (5). Within such interventions' will to improve, Li argues, also lies a ‘claim to know how other people should live, to know what is best for them, to know what they need’ (4). Yet, people may feel differently. This observation helps explain why interventions do not always unfold as planned and why they may have unexpected effects. Drawing here on, we examine the intentions behind the peer support intervention in comparison to participants' lived experiences. We find Li's concept helpful to fill in the gaps which the RCT left to explain—or as Li phrases it, the ‘gaps between what the programs proposed and what they delivered’ (123), and the gaps between the world as portrayed in interventions and the world meant to be reshaped. While a governmental rationality is well reflected upon within health intervention studies, the will to improve is not simply a ‘narrative of governmentality rising’ (32) but addresses why power ‘works positively to create new conditions, and how it is in turn shaped by the ‘strategies of struggle’ with which it is engaged in permanent relations of provocation and reversal’ (192). Taking the concept from the highlands in Indonesia to a very different setting, we find it relevant in a Danish urban context to unravel similar dynamics of the ‘messy actualities’ (283) in connection with governmental interventions. Building on the idea that the will to improve ‘falls short of the promise to make the world better than it is’ (283), we turn to experience‐near accounts of how people think and feel about it.

Combining the two analytical concepts, we explore how the experience of diabetes is framed within the peer support intervention and participants' lifeworlds, respectively. The two concepts help illuminate the tensions between the intervention's intentions and aspirations and the embodied, affective worlds of those intervened upon. Put differently, in this article, we aim to understand how the intervention failed—failed, understood not simply in terms of a good or bad result, but as a deliberate decision to learn something more from its lack of effect.

## 2 Methods

### 2.1 The Peer Support Intervention

This study was conducted through an interdisciplinary collaboration between the Department of Anthropology at the University of Copenhagen and Steno Diabetes Centre Copenhagen (SDCC), comprising two PhD projects, one biomedical and one anthropological, both exploring the use of CGM for people with T2D participating in the RCT. The RCT, lasting 12 months, included 96 participants from the SDCC outpatient clinic.

Peer support interventions vary in form (Mikolajczak‐Degrauwe et al. 2023; Joo et al. 2022). In this study, professionally led group sessions were held in a healthcare setting at SDCC, facilitated by PhD student and clinical nurse specialist in diabetes, Nanna Lind (hereafter referred to as ‘NL’), and co‐facilitated by Astrid Andrea Anesen, the first author (hereafter referred to as ‘AAA’), PhD student and medical anthropologist with expertise in diabetes, exploring participants' experiences. The peer support model was inspired by the Gillings School of Global Public Health, University of North Carolina, including five key elements: (1) the value of being there for people, (2) using people's experiences, (3) empathetic listening and encouragement, (4) linking people and healthcare professionals and (5) ongoing support (UNC Gillings School of Global Public Health 2025). Due to COVID‐19, group sizes were smaller than planned, with three groups of three participants and one of six. Each peer support group met three times, with sessions spaced two to 3 months apart, each lasting 3 hours. The sessions were structured around a dinner to create a relaxed setting. The facilitators encouraged both discussions about daily life with diabetes and emotional, technical and medical experiences with CGM. The programme was continuously adjusted according to participants' reactions. NL and AAA, as facilitators, both had previous experience conducting peer support for people with diabetes.

### 2.2 Ethnographic Fieldwork

The article is based on ethnographic fieldwork conducted by AAA from January 2020 to July 2022 as part of broader fieldwork on living with T2D and CGM. This part of the fieldwork aimed at gaining insights into how the intervention resonated with participants' needs. AAA attended all group meetings and conducted interviews. The various forms of ethnographic material supported one another and were treated equally. Audio recordings of meetings captured social interactions such as sequences of silence and casual conversation during dinner, offering insight into when participants were verbally engaged or disengaged. Participant observation, documented in field notes, highlighted bodily expressions and subtle nonverbal interactions, including eye contact, and visible engagement and disengagement. Follow‐up interviews were conducted a few weeks after participants' final peer support session using a semi‐structured interview schedule that encouraged them to describe their experiences in their own words. Interviews lasted between 30 min and 2 h and covered both positive and negative experiences, expectations, relationships with peers and the facilitators' roles. Interviews took place at participants' homes, workplaces, the diabetes clinic or via phone or Zoom because of COVID‐19 restrictions. As the interviews were part of AAA's broader fieldwork, she sometimes sensed a shift in the atmosphere when switching from questions on living with T2D and CGM to questions about peer support. The shift was observed in a changed tone of voice, reduced eye contact and shorter occasionally dismissive answers. This may have stemmed from negative emotions regarding the intervention or discomfort in criticising it directly to AAA.

All participants in this study provided informed written consent, which was separate from the RCT's ethical procedure. The names of the participants were changed to protect confidentiality. One aspect in particular that requires ethical reflection is the audio recording of the peer support intervention. This practice could be perceived as intrusive and potentially disruptive to the social dynamics. Therefore, in addition to obtaining written informed consent before participation in the intervention, participants were asked for ongoing oral consent at the beginning of each session regarding the audio recording. Although it might be considered challenging to refuse consent in a group setting, all participants were already familiar with the procedure from their previous individual fieldwork experiences with AAA, during which no one declined being recorded. The rationale for audio recording sessions was to document nuances of the meeting atmosphere that participant observation alone might not reveal. As noted, the recordings yielded valuable insights into the subtle social dynamics, including tone of voice, silences, laughter, hesitation, interruptions and moments of animated discussion, which are crucial in peer settings yet may lack in detail in field notes. The facilitators remained attentive to the potential adverse effects of this methodological choice but observed no indications that participants felt inhibited by the presence of the recording device. Participants generally appeared to speak openly during meetings, sharing vulnerable and intimate dimensions of their everyday lives, such as familial entanglements or detailed physical symptoms.

### 2.3 Participants

In Denmark, people with T2D are typically referred to hospital treatment (e.g., SDCC) due to HbA1c above 53 mmol/mol or diabetes complications (e.g., cardiovascular disease), indicating their condition is too complex for the primary care sector. A total of 20 participants from the RCT were randomised to group B (CGM and peer support), all of whom were Caucasian (see Lind et al. 2024 for recruitment details). Of these, 5 participants, all men with high HbA1c (69–129 mmol/mol) and short educational backgrounds (below undergraduate level), opted out. Therefore, 15 participants participated in the intervention, 7 women and 8 men, aged 50–77 years. They had lived with diabetes for 13–23 years, and 10 lived with a partner. Participants had an HbA1c between 58 and 87 mmol/mol, and 12 had diabetes complications. The participants had diverse educational backgrounds and occupations. One participant attended meetings but declined to be interviewed. While the group of 15 participants was not considered vulnerable in socio‐economic terms, such as education and occupation, they attended SDCC rather than primary care because of high HbA1c and diabetes complications.

### 2.4 Analytical Approach

In this article, we applied a phenomenology‐inspired approach to understand the social dynamics during the intervention. Phenomenology examines how phenomena manifest in lived experiences (Desjarlais and Throop 2011), emphasising ‘the richness of people's lives, concerns and engagement in direct and incisive terms’ (Desjarlais and Throop 2011, 97). This approach allowed us to explore participants' experiences situated within their lifeworlds.

To identify shared experiences among participants, we conducted a reflexive thematic analysis inspired by the work of Virginia Braun and Victoria Clarke (2006). We first reviewed audio recordings, transcriptions and field notes to gain an overview. Next, we refined our focus, selecting key categories and relevant citations. Discussions within our interdisciplinary research group led to the consensus‐based conclusions presented in this paper. The empirical material was rich in participants' critical verbal and nonverbal reactions towards the intervention; accordingly, we focus on the challenges, uncertainties and tensions that emerged during its implementation. The thematic analysis identified four key themes capturing participants' experiences across the groups: embodied atmospheres and lacking resonance, CGM in focus: expert guidance and hopeful futures, unreflected socio‐emotional experiences and the intervention as mooded.

The analysis is case‐oriented, providing a detailed examination of shared issues across the material (Højer and Bandak 2015). Group one was chosen as the analytical focus, as it best illustrates how participants' experiences sometimes challenged the intervention's assumptions. Here, the socio‐emotional programme was most fully executed as it became less structured over time. For example, participants' reactions to a photo activity led to its cancellation in later groups. Group one thus highlights the contrast between the intervention's will to improve and participants' lifeworlds, which is the analytical prism here. We relate the findings to narratives from across the empirical material. Group one included Freddy, 62, who had lived with diabetes for 17 years, Barbara, 73, who had lived with diabetes for 18 years and Sebastian, 67, who had lived with diabetes for 17 years.

## 3 Findings

### 3.1 Embodied Atmospheres and Lacking Resonance

Across all groups, NL and AAA as facilitators struggled to create a comfortable environment that allowed peer dynamics to develop. Encapsulated in the first meeting of group one, the tensions that suffused the atmosphere in many peer support meetings are highlighted. Bodily discomfort, reluctance and silence were evident within the peer support setting studied here. Barbara had called in sick before the day's meeting, underscoring the small group's vulnerability. The following case example illustrates participants' embodied responses, which the facilitators immediately sensed. Drawing on embodiment theory (Csordas 1990; Jackson 1983), we demonstrate how participants experienced the intervention through their bodies and how the facilitators experienced the participants' immediate reactions to the intervention. From a phenomenological perspective, ‘the living body is considered the existential null point from which our various engagements with the world—whether social, eventful or physical—are transacted’ (Desjarlais and Throop 2011, 89). Thus, our analysis of the social dynamics of the peer support intervention begins with the body.

A large table in the room held small picture cards, including ones of a winter landscape and people holding hands. After discussing data security with participants upon their arrival, the facilitators encouraged Sebastian and Freddy to pick two pictures and introduce themselves. As the conversation shifted from casual to activity, the atmosphere changed. The participants went from reclining in their chairs, laughing and making eye contact, to standing around the table in silence, staring at the pictures, unsure of where to place their hands. NL broke the silence, ‘what’s interesting is how people interpret these’. AAA smiled, adding, ‘there are so many options’. Silence then returned. After minutes of quiet, everyone had selected two pictures. ‘Who wants to start?’ NL asked. After a pause, Sebastian volunteered, sharing his interest in sports and Greek mythology, glancing between his pictures and NL. Freddy spoke about his love for travel, also making eye contact with NL. NL asked follow‐up questions about their stories, but their stories were brief. After the introductions, the discussion shifted to COVID‐19 and vaccinations. Participants became more engaged and seemed more at ease.

(Meeting 1)

Atmospheres are often easier to feel than to describe in words (Schnegg 2025). Similar to many other activities, the picture card activity frequently failed to achieve genuine participant engagement. As Freddy noted in the joint evaluation at the final meeting, ‘there was never really a discussion between us but rather through you as facilitators’. In stark contrast to other social interactions, such as casual chitchat, their disengagement during less meaningful activities was often also reflected in the atmosphere. Although not verbally expressed, hesitation, uncertainty, silence and reluctance recurrently pervaded the atmosphere in meetings. In many meetings, it was subtly revealed through the way participants looked down or away, the long pauses that occured when the facilitators asked questions they did not know how to respond to, their avoidance of eye contact or their tendency to focus on the facilitators when speaking. In another group, the circumstance that a participant in particular found many activities unmeaningful was evident in bodily reactions such as glances at the phone and subtle sighs of boredom, and, more explicitly, attempts to move on from unengaging activities. This was sensed by the other participants and had a significant impact on the overall atmosphere. Schnegg (2025) proposes emotions as atmospheres; ‘in situations, people, objects, practices and other beings connect and constitute atmospheres that touch people emotionally’ (5). Participants' (embodied) reactions to the activities introduced in the intervention often shaped the situation, creating a particular tonality or a sense of ‘something in the air’ (11), which was often accompanied by disengagement and disapproval palpable in the room, felt and shared by the people present.

The picture card activity conveyed something more about the participants' lifeworlds: diabetes, it seemed, was deliberately not mentioned in their narratives unless directly asked about it. Across groups, participants chose picture cards representing activities they enjoyed—such as sailing, walking in the woods, sewing and gardening—highlighting aspects of daily life that made them happy. When asked to reflect on how diabetes affected their everyday lives, participants often expressed uncertainty about the purposefulness of such questions. Responses like ‘I don't know how to respond’ or ‘nothing comes to mind’ were common during the socio‐emotionally focused activities.

Rosa (2019) introduces the concept of resonance as a relational phenomenon, embedded in atmospheres. Resonance is both bodily and cognitive, representing a meaningful exchange between subject and world. Resonance thus requires active engagement. A passage from the same meeting illustrates this lack of resonance among participants. When the facilitators briefly left to prepare dinner, they provided questions for the participants to reflect on, such as ‘what would you like people without diabetes to know?’ ‘What is difficult about living with diabetes?’ and ‘What works well in your life with diabetes?’ As they left, Sebastian remarked, ‘I haven't discussed such matters with others in many years’.

‘Now let’s hear what you wrote down earlier. Did you discuss your thoughts?’ NL asked after dinner. ‘No, not really’, Sebastian replied. ‘No, not directly, we talked about other stuff’, Freddy added. ‘But I wrote down that others should know that you can live a completely normal life with diabetes. Many people believe it’s something unusual, but it’s not. With type 1 diabetes, it’s a significant part of your life, but with type 2 diabetes, most of us can forget about it. I’ve never felt anything, not even highs or lows (glucose values)—maybe slight restlessness when really low. The only thing is I’ve started to feel a tingling in my feet. Other than that, I never really notice it. In general, it’s an ordinary life’, Freddy explained.

(Meeting 1)

Although the peer support intervention aimed to create a space for discussing otherwise overlooked everyday life issues with peers, many participants, such as Freddy, preferred to maintain an ordinary life and consequently did not experience a particular or unmet need in daily life that peer support could address. In the above excerpt, Freddy acknowledged the challenges of diabetes self‐care and its potential long‐term consequences, but he downplayed their significance. Rather than engaging in discussion, he concluded that life with diabetes was no different from that of everyone else. Towards the end of the same meeting, the facilitators asked what Freddy and Sebastian wanted to discuss next time, reminding them this was a space to explore diabetes's impact beyond clinical settings.

‘I’ve moved past that. I’ve had diabetes for years, so I’m over it’, Sebastian said. Freddy looked thoughtful for a moment and said, ‘some of these things, you seem to be fishing for—it’s nice that you can discuss various topics—but with so much information online, if you're unsure about something, you simply Google it. You don't spend a long time wondering’.

(Meeting 1)

Similar to Sebastian and Freddy, many participants did not recognise the intervention's premise of an unmet need for discussing everyday life issues with peers. This was also linked to their motivations and expectations within the RCT framework. One participant told AAA during the interview that he had signed up explicitly for the peer support intervention, saying, ‘it's nice to get the opportunity to get out of the house, meet other people and have dinner together’. However, most participants were primarily motivated by access to the CGM technology, rather than, it seemed, socio‐emotional support. A participant politely noted to AAA in the interview, ‘it hasn't bothered me to participate in these meetings, except that I had to spend a few evenings on them. That's nothing compared to getting to participate in the trial for an entire year’. For many participants, it appeared, participation in the peer support intervention was a trade‐off for the CGM technology.

### 3.2 CGM in Focus: Expert Guidance and Hopeful Futures

CGM frequently emerged in discussions, both initiated by the facilitators and participants, engaging everyone across the groups. However, participants had specific preferences for how they wanted to discuss it. A phenomenological approach to experience emphasises how our existence is inherently temporally structured (Desjarlais and Throop 2011). Recognising that people make sense of the world through time, we examined CGM in light of its capacity to shape futures and its connotations of hope and potential. Despite numerous failed attempts at peer discussions, there were moments of enthusiasm, often linked to the use of CGM. At their second meeting, Sebastian, Freddy and Barbara were already deep in conversation about their CGM use, before AAA even turned on the recorder:

‘I’ve experienced that too, sometimes the glucose is just out of control’, Sebastian lamented. ‘Exactly’, Barbara exclaimed. ‘Sometimes if my glucose is high, it drops so slowly, even overnight it hasn’t come down. At other times, it falls quickly within the first few hours of sleep. I can’t make sense of it, and it’s something you’ve never seen before with the finger pricker’, Freddy added. ‘So, you've all been surprised by what CGM reveals?’ AAA asked, supporting the discussion while NL was home, sick. ‘Yes’, Sebastian and Freddy confirmed in unison. ‘I'm so glad I don't have to prick myself. I have sensory disturbances in my hands. This device is a huge help’, Barbara elaborated. ‘It’s simply great’, Freddy agreed. The atmosphere was filled with enthusiasm.

(Meeting 2)

This social interaction highlights how CGM occasionally prompted lively discussions across all groups. In this instance, without much probing, a meaningful conversation about their experiences with CGM effortlessly unfolded between the peers. While both facilitators were away to get dinner during meetings, participants occasionally exchanged CGM experiences during their time alone. In one group, a participant said, ‘I'm about to go crazy from the constant alarms,' while another shared, ‘I changed the settings, so alarms go off above 12 instead of 10 to be more realistic’. This exchange sparked a broader discussion about individual CGM settings. Often, the CGM alarms would sound around the table after dinner, interrupting conversations and drawing participants' attention. In connection with the casual dinner, it sometimes started peer discussions on how different foods affected the glucose levels, such as when one participant asked another, ‘have you overeaten?’ replying, ‘indeed, I have’.

Many meetings began with the facilitators asking about CGM, such as whether anyone had new experiences with the technology. However, contrary to the above examples of peer discussions, when NL was present, participants often took the opportunity to ask her individual and technical questions. Questions related to CGM could concern the connection between exercise and glucose levels, now visualised with CGM, guidance on individual device settings or they could be about living with diabetes in general, such as how stress affects glucose levels in biomedical terms. Referring to the education on CGM use they had received as part of the RCT, participants often referred to NL in her role as a teacher and healthcare professional. During the follow‐up interviews, many participants highlighted how they would have preferred the opportunity to ask NL more questions or more education on CGM use. Unlike similar or dissimilar experiences with peers, they often sought her expert knowledge. One participant was particularly eager to have his CGM data uploaded and displayed on a screen during a meetingand suggested that participants could take turns having their data displayed on a computer screen and evaluated by NL. Although the facilitators appreciated the enthusiasm, the idea was deemed unsuitable for the context of peer support, as it involved activities typically conducted during clinical visits. Consequently, while the topic of CGM motivated advice‐seeking and inspiration, it was often approached in the form of individual guidance and technical care. One participant said, ‘NL gave me the real nuggets of gold’. Another added, ‘when first diagnosed with diabetes, you get a lot of information, but that was a long time ago, so I found what NL shared interesting’. For many, CGM felt like a new beginning in their diabetes experience, requiring fresh information and professional guidance, much like at the time of their initial diagnosis.

A few weeks after the final peer group meeting, AAA interviewed Sebastian regarding his experience. He reflected:

‘The meetings were mostly about the social—we talked about how we felt. Nothing new happened in the last two meetings; we didn’t receive new information’, Sebastian began. ‘I would have preferred a greater focus on actions, that is, new things we can try, and the latest treatment. I probably expected us to have discussed more about what I gain from constantly monitoring my glucose with the sensor. Now mine are pretty high’, he elaborated. Sebastian went on suggesting, ‘you could have involved a doctor or a dietitian, so that everything was in one place. Alternatively, we could have gone to the gym to see how we reacted to the exercise, and then NL could explain why that is afterwards’.

(Interview)

For Sebastian, the intervention would have been more meaningful had it focused on practical strategies rather than emotions and social aspects. He was more interested in information about how CGM could help him lower his glucose levels and treatment options. While socio‐emotional issues felt beyond his control—and issues he wanted to move past—the CGM represented a promising future, making it a more appealing topic. In another group, two participants felt their third group member was too ‘pessimistic’, sharing only her ‘despair’ about living with diabetes. ‘Whenever she spoke, we all had to recover and take a deep breath. There was never really room for any constructive communication’, one participant explained, describing how it affected the social dynamics. The two participants wanted to focus on ‘moving forward’ in life with diabetes and requested instead that the facilitators provide ‘education’ on CGM use to enhance their situation. Ultimately, the intervention's will to address participants' socio‐emotional challenges often clashed with participants' lifeworlds, as many sought practical guidance and expertise on how to improve their glucose levels through CGM use rather than socio‐emotional support, which the intervention failed to provide them sufficiently.

### 3.3 Unreflected Socio‐Emotional Experiences

As shown, the intervention's activities addressing socio‐emotional needs often failed to engage participants actively. Drawing on different modes of reflectiveness in existence, we explore how these issues appeared deeply embedded in their lifeworlds, making it difficult to approach them. During the second meeting, after the spontaneous discussion on CGM addressed above, the facilitators decided to move the conversation forward by introducing a diabetes board game. The game is designed to encourage discussions about the challenges of living with diabetes and has been tested with groups of three to five participants, yielding positive feedback from participants (Stenov et al. 2021). The social interactions surrounding it are exemplified by case one below.

‘What do you say?’ AAA asked Barbara, Sebastian, and Freddy after introducing the game. They nodded but said nothing, and each selected a persona in silence. Barbara’s character, Pia, felt consumed by worries about the future due to diabetes. Asked if she related, Barbara replied firmly, ‘I don't worry in my daily life’. Freddy’s persona, Jens, led a busy life and did not feel his diabetes, but was concerned about diabetes complications. Freddy reflected, ‘I also have a busy life and don’t feel my diabetes, except for a tingling in my feet, but it’s not something I think about. However, I am worried about diabetes complications. You don’t live exactly as before, but you try to. I think you should be able to enjoy life and do whatever you like’. Sebastian’s character, Annette, struggled with her weight and wanted to eat healthier and start exercising, but lacked the time. ‘I want to eat healthier, but my wife handles that’, Sebastian said. ‘I also have grandchildren, and they stop by and say, ‘granddad, let’s do something today’. Now I have the time, I didn’t five years ago. I walk the dog and bicycle for 1.5 hours. I am freer now and have fewer work assignments’, he explained. The game was paused to eat dinner. ‘It would be a loss if we never got to finish the game. That would make me so sad’, Freddy said jokingly, and all laughed.

(Meeting 2)

Although Barbara, Freddy and Sebastian agreed to play the diabetes board game, they did not seem to find it entertaining or valuable. During the game, they appeared quieter, and there were few peer discussions. They mostly spoke when prompted. As illustrated in the paragraph above, participants' narratives of not worrying, not feeling their diabetes, enjoying life and feeling freer during a game about diabetes challenges are striking. Similar to Barbara, Freddy and Sebastian, many participants seemed to share their reluctance towards the game. A few participants reported enjoying it, describing it as ‘entertaining’ and ‘sparking conversations’. However, most participants considered it a ‘waste of time’ and ‘irrelevant’, with some even finding it ‘childish’ and ‘annoying’. By tracing the intervention's lived effects, we see that its aim to address participants' socio‐emotional well‐being alienated those who found it difficult to relate to the activities. The intervention's will to improve was misaligned with participants' needs and wants, eliciting negative emotions regarding the game, which was often the case. Notably, while the game was tested by people who volunteered to play it (Stenov et al. 2021), participants in this study volunteered for CGM testing and were assigned to peer support through the RCT.

The same lack of meaningfulness among participants was also evident in a photo activity, as demonstrated by the following example. The participants were asked to bring one or two pictures from their everyday lives with diabetes. This could be anything, it was noted, a situation where the CGM was helpful or annoying, a situation where diabetes was especially present or the opposite. Most participants did not bring pictures, explaining this as forgetting the task (despite text message reminders) or not knowing what to photograph.

‘Did you bring pictures?’ AAA asked the participants. Both Freddy and Sebastian looked away, not meeting the gaze of the facilitators. Neither of them had brought any. ‘I did think about it quite some time’, Freddy said after a little while. ‘I looked through my pictures and thought, ‘what’s a picture of how to live with diabetes?’ I asked my wife, ‘how do we live with diabetes?’ I don’t know. I asked, ‘what’s wrong with how I live?’ I don’t do anything special because I have diabetes. It’s in your head. So, I can't seem to think of anything I do differently that I could take a picture of. My wife said, ‘take a picture of you eating a salad bowl’. But all people do that; there’s nothing special about that’, Freddy reflected.

(Meeting 2)

Whereas participants expressed concerns, such as Freddy's worries about diabetes complications, the activities never fully captured the sometimes conflicting illness experience. For Freddy, socio‐emotional issues related to his diabetes existed primarily in his mind, and the activities failed to bring these concerns into the peer support setting. From a phenomenological perspective, existence is characterised by different modes of engagement, with social actors adopting more or less reflective attitudes towards their experiences (Csordas 1994; Jackson 1996; Desjarlais and Throop 2011). Socio‐emotional issues appeared to be deeply ingrained in participants' lifeworlds, existing in an unreflected form. Being asked to engage in such intensely personal aspects of life made participants feel uncomfortable and awkward, as they could not relate to these issues within the context of the intervention. In contrast, CGM, as a new element in their daily lives, seemed to exist in a more reflective mode, making it easier for participants to engage with.

### 3.4 The Intervention as Mooded

Addressing the peer support intervention as a mooded experience revealed more profound insights into the participants' lifeworlds and their attitudes towards the intervention's activities. According to Throop (2017), ‘moods may illuminate something about the world (or worlds) into which particular individuals find themselves thrown’ (199–200). He further explains that ‘moods are often complex, heterogeneous and dynamic existential attunements to the world that often mix with other thoughts, images, fantasies, strivings, desires, emotions and affects’ (Throop 2017, 201). From this perspective, participants' negative responses to the intervention's activities, both explicit and implicit, were ‘mooded’, as their illness narratives reflected assumptions about a good life with diabetes. This is one explanation for why the peer support activities often conflicted with participants' own experiences, leading to adverse reactions. These conflicts could emerge when participants distanced themselves from board game characters facing challenges, or when they were asked to take pictures, thus forced to foreground living with diabetes. Many participants, more explicitly, shared opposing experiences of how diabetes was framed in the intervention during the follow‐up interviews. The following exchange between Freddy and AAA illustrates this dynamic:

‘I usually don't tell people I have diabetes, but here, we all knew, so you didn't have to think about it. This made it easier to open up’, Freddy said. ‘So, did you discuss things in the meetings that you wouldn’t elsewhere?’ AAA asked. ‘Yes, you’ve heard about other people's everyday situations, and I shared some of my own, I wouldn't tell others’, he replied. ‘Did you find that valuable?’ AAA asked. ‘No, not for me. I don’t feel the need to discuss diabetes with others’, Freddy explained.

(Interview)

Freddy acknowledged that the peer support intervention allowed for intimate sharing about life with diabetes. However, he, like many others, did not find these discussions meaningful. Barbara's experience further illustrates this. As for Barbara, participants' mooded experience of illness also influenced their attitude towards the peer support intervention. A few weeks after her final meeting, AAA visited Barbara. Barbara called in sick before a meeting due to nausea and dizziness from the medication, which she had now stopped using. Recalling a discussion about the medication during the diabetes board game, AAA asked whether Barbara had found it helpful.

Barbara reflected, ‘many times, it does more harm than good because you end up feeling sorry for yourself, and you think, you’ll get the same symptoms. But medication affects people differently. If I had just listened to how badly he felt, I could have felt bad mentally, too. But I thought, ‘no, I'll give it a shot.’ I don't want to sit and listen to other people's pity’. AAA asked, ‘what does that do to you?’ ‘It puts me in a bad mood. You hear some terrible stories. I'd rather immerse myself in a good book. There must be more to talk about than just illness’, she explained. ‘That doesn't help you?’ AAA interjected. ‘I feel like this’, Barbara went on. ‘If I'm feeling bad one day, I don't take it out on Thomas (Barbara’s husband), because he has his own struggles. I don't do that to my family either. They can see if I feel bad one day, but you shouldn't let illness overwhelm you’.

(Interview)

Barbara shared how she disliked talking about illness, as it put her in a bad mood. Instead, she preferred to immerse herself in activities that distracted her from health concerns and opened up other worlds. Many participants emphasised the importance of engaging in normal daily activities to cope with their illness. In contrast, the intervention kept them focused on a ‘diabetes world’, centring on diabetes‐related concerns and challenges. Barbara did not confide in her husband or family about her worries, wanting to spare them the emotional burden of her struggles. With illness already a significant part of her life, her and her husband's diabetes, along with other health issues, she deliberately focused on things that made her happy to avoid feeling overwhelmed. Similarly, Barbara found the peer discussions about medication unhelpful. Many participants shared this perspective within the context of the peer support intervention. In one group, they agreed that they rarely thought about diabetes in their everyday lives and did not need to discuss it with their peers. ‘Life is too short for that’, one said. Another participant, almost provoked, remarked, ‘some of those questions about living with diabetes, I could throw up’. Thus, many participants distanced themselves from ‘people who wanted to talk about illness all the time’, as one participant put it. Another participant explained, ‘we know some people, we avoid them because they always talk about their illnesses, and I don't want to tell them about mine’. A participant emphasised, ‘diabetes should not define you’. To maintain a positive outlook, participants deliberately adopted an optimistic mindset to stay mentally afloat in their daily lives, narrating their illness experience differently than the intervention framed it. The intervention's focus evoked negative thoughts and emotions tied to their diabetes experience. One participant described it as ‘navel‐gazing’ during the joint evaluation at the last meeting and admitted, ‘getting annoyed with those who complained about the challenges of managing their diabetes’. Illuminated was an ‘atmospheric‐like spread of moods’ (Throop 2017, 199) felt by participants within the context of the intervention, resulting from imaginings of a good life with diabetes, contrasting with what the intervention's activities brought to the fore for participants as a collective.

## 4 Discussion

Approaching failure as a means of learning, we sought to understand why the peer support intervention did not engage participants. Medical anthropologists and sociologists have criticised public health interventions for assuming a linear cause‐and‐effect relationship (Gibson et al. 2022; Yates‐Doerr 2020). Such interventions, aiming to address causal factors, often ignore ‘sociality and the complex and relational ways in which health is made, lived and done’ (Gibson et al. 2022, 1150). It became clear that peer support is a ‘complex intervention that takes place within a personal journey of health and illness’ (Walker and Peterson 2021, 224).

By emphasising the socio‐emotional aspects of living with diabetes, the intervention may have neglected other critical elements. Participants did not find the intervention's focus relevant in isolation. As Mol (2006) argues, health concerns should not be treated separately but as interconnected elements, ‘disease, illness, technology, treatment and life: they come as a package, so it would be better to study them in this way’ (412).

Health interventions are not neutral solutions; they shape the very problems they aim to address (Frohlich and Potvin 2008; Koch and Svendsen 2005; Høybye et al. 2023). As Emily Yates‐Doerr (2020) notes, attempts to ‘fix’ an issue can reinforce the very problem they seek to solve. As expert interventions identify deficiencies to be fixed through expert knowledge, says Li (2007), the will to improve is ultimately converted into the practice of ‘problematisation’. In this case, the intervention framed diabetes as a socio‐emotional problem, thereby implicitly claiming to know how participants should experience and feel about their illness. In the name of expert knowledge, the object for improvement becomes visible and open to intervention, Li argues. This placed the improvement and fixing of participants' socio‐emotional self‐care practices at the centre of the intervention. In turn, Li emphasises moving beyond the intervention's inherent logic to understand how interventions are lived by people whose lives they aim to improve. Comprehending how the peer support intervention unfolded in real life involved illuminating the diabetes experience as framed as being at odds with participants' lifeworlds. The intervention presented them with a version of their lifeworlds in which values and priorities were shifted, so that for them meaningless issues came to be highlighted through the activities. Participants distanced themselves from the intervention's problematising and fixing, which did not resonate with their lifeworlds. This tension made participants feel uncomfortable or uninterested during many activities. Li notes, there are ‘moments when the targets of expert schemes reveal, in word or deed, their own critical analysis of the problems that confront them’ (11), leading to ‘prickly subjects’, seen in participants' de‐problematisation of life with diabetes. In the social context explored here, the participants sought to keep challenges of living with chronic illness in the background, whereas foregrounding hopeful aspects of their lives. The intervention thus yielded participant perspectives on joyful activities beyond the diabetes world in the pursuit of a good life, in particular. They engaged in discussions about their futures with CGM, with which they identified, while bodily and verbally manifesting their critique towards the socio‐emotionally focused activities.

A key observation is how participants' limited need for socio‐emotional support contributed to the intervention's lack of meaningfulness. The intervention's design did not align with participants' actual needs, suggesting that those with an unmet need for such support may have benefited more. Participants chose to trial CGM but did not actively opt into peer support, creating a disconnect between their expectations and motivations and the peer support framework. This misalignment shaped the intervention's outcomes, as the anticipated benefits of peer support did not occur in the context where it was neither sought nor prioritised. Although most participants experienced diabetes‐related complications, they had access to comprehensive clinical care through the Danish healthcare system. Participants thus led rather ordinary lives, especially when compared to the conditions of living with diabetes from a global perspective (Gammeltoft et al. 2022; Mendenhall 2019; Moran‐Thomas 2019; Weaver 2019), which potentially reduced their need for support. Yet, the intervention's activities occasionally revealed participants' socio‐emotional concerns, such as worries about diabetes complications; however, the intervention setting did not turn them into topics for discussion. This, we have argued, was due to the intervention's problematising of diabetes. Participants may have found it more useful if the intervention had been framed differently—more hopeful and more open.

NL and AAA, as facilitators, occupied more space in meetings than anticipated with the small groups, demanding critical self‐reflection on their positioning. Conducting the intervention as part of their PhD projects, they were already familiar with the participants. Whereas this familiarity may have provided reassurance, it also contrasted with the reciprocity among peers. AAA, who had type 1 diabetes, a similar yet distinct condition, rarely discussed her illness, though participants were aware of it. NL, a healthcare professional, was inevitably perceived as such and often drew on her clinical expertise. These study conditions, among others, likely influenced the social dynamics and, by extension, the outcome of the intervention. The conclusions could have been different if other facilitators had conducted the intervention, underscoring the influence of facilitator–participant dynamics.

## 5 Conclusion

This ethnographic study examined the reception of a peer support intervention in Denmark by its participants. The study adds nuance to our understanding of the social dynamics of how health interventions work and how they fail, providing insights to inform research beyond healthcare to other fields where interventions are implemented, such as criminology or environmental initiatives. Instead of minimising or writing out the failures, we have insisted on failure as a dimension of potential. A great deal can be learnt from viewing failure as productive. A central finding in this qualitative study, not captured in the RCT's quantitative outcome measures, was how the intervention's framing of participants' socio‐emotional lives did not resonate with their lifeworlds. The lessons learnt are helpful when designing future health interventions. Combining the concepts of lifeworld and the will to improve, we have approached the social dynamics within a peer support intervention. This way, we captured how people with T2D discussed navigating their everyday worlds with peers, including disruptions brought on by illness or intervention. We explored how, when interventions interact with people's embodied lifeworlds, they may align with their values and experiences, or they may fail to do so. Through ethnographic accounts of the intervention, we have asked how such social initiatives are received, negotiated and resisted, and what this reveals about the conditions under which health interventions come to feel meaningful or not. In contrast to the will to improve, we propose a ‘lifeworlding’ approach to future health interventions. By lifeworlding health interventions, we mean the process of attuning their aims, activities and measures of success to the everyday lived experiences—the lifeworlds—of participants. It is not simply contextualising care but reshaping the intervention. Rather than imposing an external framing of problems and solutions, lifeworlding embeds interventions within the textures of participants' own meanings and priorities. Conversely, non‐lifeworlded interventions risk misalignment, in which the intervention's own logic sidelines participants' priorities. Unlike generic calls for ‘patient‐centred care’, lifeworlding emphasises not only taking participants' perspectives into account but also recognising when the intervention's very framing of the problem is at odds with those perspectives, and being willing to reconfigure aims and methods accordingly.

## Author Contributions

**Astrid Andrea Anesen:** study design (equal), conceptualisation (equal), methodology (equal), investigation (lead), project administration (lead), resources (lead), curation – transcription and coding (lead), formal analysis (lead), validation (equal), writing – original draft preparation (lead), writing – review and editing (equal). **Bryan Cleal:** study design (equal), methodology (equal), supervision (equal), formal analysis (supporting), validation (equal), writing – review and editing (equal). **Tine M. Gammeltoft:** conceptualisation (equal), methodology (equal), supervision (equal), formal analysis (supporting), validation (equal), writing – review and editing (equal).

## Ethics Statement

The RCT was approved by the Danish Data Protection Agency (J‐2020‐100) and the Scientific Ethics Committee of the Capital Region, Denmark (H‐20000843). The RCT is enlisted at Clinicaltrials.gov (NCT04331444).

## Consent

All participants in the qualitative study gave informed written consent (separate from the RCT ethical procedure). The names of the participants have been changed in the article to protect confidentiality.

## Conflicts of Interest

The authors declare no conflicts of interest.

## Data Availability

The ethnographic material supporting this study's findings are available upon reasonable request from the corresponding author. Restrictions may apply to access because of ethical considerations and concerns about protecting participants' anonymity.
